# Analytical Investigation of the Time-Dependent Stagnation Point Flow of a CNT Nanofluid over a Stretching Surface

**DOI:** 10.3390/nano12071108

**Published:** 2022-03-28

**Authors:** Ali Rehman, Anwar Saeed, Zabidin Salleh, Rashid Jan, Poom Kumam

**Affiliations:** 1 Department of Mathematics, Faculty of Ocean Engineering Technology and Informatics, Universiti Malaysia Terengganu, Kuala Nerus 21030, Terengganu, Malaysia; alirehmanchd8@gmail.com (A.R.); zabidin@umt.edu.my (Z.S.); 2 Center of Excellence in Theoretical and Computational Science (TaCS-CoE), Science Laboratory Building, Faculty of Science, King Mongkut’s University of Technology Thonburi (KMUTT), 126 Pracha-Uthit Road, Bang Mod, Thung Khru, Bangkok 10140, Thailand; 3 Department of Mathematics, Univesity of Swabi, Swabi 94640, Pakistan; rashid_ash2000@yahoo.com; 4 Department of Medical Research, China Medical University Hospital, China Medical University, Taichung 40402, Taiwan

**Keywords:** CNTs, nanofluid, stretching surface, heat transfer, stagnation point

## Abstract

The heat transfer ratio has an important role in industry and the engineering sector; the heat transfer ratios of CNT nanofluids are high compared to other nanofluids. This paper examines the analytical investigation of the time-dependent stagnation point flow of a CNT nanofluid over a stretching surface. For the investigation of the various physical restrictions, single and multi-walled carbon nanotubes (SWCNTs, MWCNTs) were used and compared. The defined similarity transformation was used, to reduce the given nonlinear partial differential equations (PDEs) to nonlinear ordinary differential equations (ODEs). The model nonlinear ordinary differential equations were solved, with an approximate analytical (OHAM) optimal homotopy asymptotic method being used for the model problem. The impact of different parameters such as magnetic field parameter, unsteady parameter, dimensionless nanoparticles volume friction, Prandtl number, and Eckert number are interpreted using graphs, in the form of the velocity and temperature profile.

## 1. Introduction

The study of Magneto–Marangoni convection, which is caused by surface tension, has been a popular research topic for scientists and engineers in recent years. This is due to the numerous applications, including thin liquid layer scattering, atomic reactors, semiconductor processing, dynamic uses in the welding process, crystal development, material science, varnishes, silicon melting, and many more. In addition, the Marangoni phenomena are commonly exploited in fine art mechanisms, such as pigment on the ground. The colorant or dye is suspended on the outside surface of the needed medium, such as water or another thickness fluid, in this technique. To make a print, the medium is encased in rag or paper. Pop et al. [1] investigated the various characteristics of the Marangoni convection technique for thermo-solutal boundary films. Marangoni convection was researched by Al-Mudhaf and Chamkha [2], utilizing a temperature and solute gradient moving through a porous medium. Wang [3] used perturbation solutions to implement the Marangoni convection concept in a thin film spray. By examining the power-law model under Marangoni, Chen [4] extended Wang’s idea. Magyari and Chamkha [5] used the flow assumption of a high Reynolds number to study the influence of Marangoni convection. Lin et al. [6,7] investigated the architectures of a MHD Marangoni-convective exchange of power-law nanoliquid with temperature gradients in a MHD Marangoni-convective exchange of power-law nanoliquid. Aly and Ebaid [8] used the Laplace transformation on a permeable surface and convective boundary film conditions, to compute the exact solution of Marangoni flow of a viscid nanoliquid. Ellahi et al. [9] studied the effects of various forms of nanoscale materials in an ethylene glycol-based aqueous nanofluid solution. The analytical results of Marangoni convective heat conversation of power-law liquids in permeable media were computed by Xu and Chen [10]. Sheikholeslami [11] proposed an ideal model of two-phase Marangoni convective MHD nanoliquid hydrothermal flow. Various types of research have recently focused on the Marangoni convection flow in various flow features [12,13,14,15,16]. 

The energy crisis has become a major topic in recent years, with many academics attempting to discover advanced resources for restoring energy and controlling the consumption of heat transfer devices. Solids, gases, and liquids are all part of these resources. The addition of tiny (1–100 nm) solid metal particles into common liquids can improve their thermal efficiency. Working liquids with low heat transfer rates, such as bioliquids, polymeric solutions, various oils, greases, water, toluene, refrigerants, ethylene glycol, and others, are widely utilized in a variety of engineering and scientific applications. The well-known metallic entities and their oxides, ${\mathrm{TiO}}_{2}{\mathrm{Al}}_{2}O_{3}$, are placed in operating liquids to improve the thermal efficiency of these liquids. Choi [17] established the optimal theory of nanoscale metallic object dispersal in molecular fluids from this perspective. He conducted an experiment on nanoliquids and came to the conclusion that the contribution of these metallic items is a fantastic means for improving thermal proficiency. After combining nanoparticles of Cu at a rate of 0.3 percent volume fraction, Eastman et al. [18] observed a 40% improvement in the thermal performance of $C_{2}H_{6}O_{2}$. Khamis et al. [19] investigated the heat transport of ${\mathrm{Cu}}_{2}{\mathrm{Al}}_{3}+H_{2}O$ and nanoliquids through a porous pipe. Using ${\mathrm{Al}}_{2}O_{3}$ water nanoliquids, Malvandi and Ganji [20] investigated the effect of nanoparticle volume fraction on heat transfer rate improvement. Using the ${\mathrm{Al}}_{2}{\mathrm{OAg}}_{3}+H_{2}O$ nanoliquid flow through an upright cone, Reddy and Chamkha [21] discovered a significant increase in the rate of heat exchange. The movement of ${\mathrm{Al}}_{2}O_{3}$-based non-Newtonian nanoliquids over a circular region was studied by Barnoon and Toghraie [22]. A study of nanofluid flow in a microchannel was completed by Hossenine et al. [23]. Carbon nanotubes (CNTs) are carbon tube-shaped particles of nanometer dimensions. The strong connection between carbon atoms distinguishes CNTs, and the tubes can have a high aspect ratio. CNTs are classed as single-walled or multi-walled nanotubes (SWNTs or MWNTs), depending on their structure. Different properties of CNT nanoparticles, such as physical, electrical, optical, and thermal, have boosted the use of nanofluids in engineering processes such as nano- and microelectronics, biosensors, ultra-capacitors, atomic reactors, gas storage, textile engineering, flat-plate display, and medicinal tools. 

Nonporous cleansers, solar collectors, and a variety of coatings are among the many other applications for CNTs. The flow of a nanoliquid containing nanoparticles was discussed by Xie and colleagues [24] (MWCNTs). The thermal characteristics of nanoliquids were improved using these nanoparticles. The motion and heat transport properties of CNT nanofluids over a tube were studied by Ding et al. [25]. Haq et al. [26] used a stretching plate to calculate the numerical results of three distinct molecular liquid flows incorporating CNT nanoparticles. When the volume fraction of particles was 15%, Ueki et al. [27] found that the improvement in thermal properties of carbon blocks increased by 7% and nanopowder increases by 19%. The 3D flow of both nanoparticles (SWCNTs/water and MWCNTs/water) and motor oil was studied by Rehman et al. [28]. The numerical research of CNT–nanoliquids flow and heat transport through a perpendicular cone was developed by Sreedevi et al. [29]. The flow of SWCNT- and MWCNT-nanofluid with a revolving and extending disc was studied by Jyothi et al. [30]. In this respect, a number of researchers [31,32] have recently looked at the flow of CNT nanoparticles from various physical perspectives. Due to their presence in a variety of technical and engineering applications, such as cylinder surface patterning [33], the cooling of optical fibers [34], greasing films on the inner walls of tubes, and condensing vapor on heat pipes [35] have been thoroughly investigated. Frenkel [36] pioneered modelling research on liquid film flow on a cylinder surface. Different models have been investigated to analyze the falling of a liquid film on a perpendicular cylinder, including the thin liquid model [37] and the thick film model [38]. The experimental examination of thin fluid film motion on a vertical cylinder by Duprat et al. [39] revealed that four different forms of flow can occur in the scheme. The 3D flow of a thin liquid sheet on a vertical cylinder was studied by Ding and Wong [40]. Alshomrani and Gul [41] investigated a thin film spray of an ${\mathrm{Al}}_{2}{\mathrm{OCu}}_{3}$ nanoliquid over a slick cylinder. Ali et al. [42] investigated the CNTs nanofluid across a stretched surface utilizing blood as a base fluid. The nonlinear dynamics of thin fluid flows through a hot cylinder were studied by Chao et al. [43]. 

Inspired by the literature, we present an analytical investigation of time-dependent stagnation point flow of a CNT nanofluid over a stretching surface. The key objective of this analysis was to improve the heat transfer ratio, because heat transfer ratio has some important applications in the engineering and industrial sectors. In our research paper, we used a CNT nanofluid, which has a higher heat transfer ratio compared to other nanofluids; we also discuss the convergence of the approximate analytical method and the effects of different parameters on fluid particle motion, as well as a comparison of the present research work with the previously published work. The flow analysis is considered over a stretching surface; the similarity transformation is used to convert the nondimensionless form of the differential equation to the dimensionless form. The following characteristics indicate the study’s novelty: A uniform magnetic field was used to study the approximate analytical solution of the thin layer flow of a nanofluid. This was the first investigation of the analytic solution of this model. Liao et al. [44,45,46] found an approximate solution technique that does not depend upon the small parameters used to discover the problem’s series solution. Using the BVP 2.0 program, the residual sum of the acquired results was calculated up to the 25th order approximation [47,48,49,50,51].

## 2. Mathematical Formulation 

Consider the time-dependent 2D stagnation point flow of a CNT nanofluid along with heat transfer in the presence of a magnetic field on a stretching surface. The continuity, momentum, and temperature equation for the given flow problem is given below:

(1) $$\frac{\partial u}{\partial x}+\frac{\partial v}{\partial y}=0,$$

(2) $$\rho_{nf}\left(\frac{\partial u}{\partial t}+u\frac{\partial u}{\partial x}+v\frac{\partial u}{\partial y}\right)=\left(U_{s}\frac{dU_{s}}{dx}+\mu_{nf}\frac{\partial^{2}u}{\partial y^{2}}-\sigma_{nf}B_{0}^{2}\left(u-U_{s}\right)\right),$$

(3) $$\frac{\partial T}{\partial t}+u\frac{\partial T}{\partial t}+v\frac{\partial T}{\partial t}=\frac{k}{{\left(\rho C_{p}\right)}_{nf}}\frac{\partial^{2}T}{\partial y^{2}}+\frac{\mu_{nf}}{{\left(\rho Cp\right)}_{nf}}{\left(\frac{\partial u}{\partial y}\right)}^{2}$$

T represents temperature of the surface, k represents the thermal conductivity, ρ represents the density of the fluid, and $c_{p}$ represents the specific heat. u and v are the velocity component along the x and y directions, the distance along the sheet is denoted by x  and the distance perpendicular to the sheet is denoted by y, the velocity of the stagnation point is denoted by $U_{w}=\mathrm{ax}\;\mathrm{with}\;a\;>0$, ν represents kinematic viscosity of the fluid.

The boundary condition for velocity and temperature are

(4) $$\begin{array}{l} u=U\;{}_{w},\;v=0\;at\;y=0;\;u\;\to U\;{}_{s}\;as\;y\to \infty ,\\ T=T_{\;w}\;at\;y=0;\;T\;\to T_{\infty }\;as\;y\;\to \infty . \end{array}$$

$T_{w}$ represents the temperature of the sheet, and $T_{\infty }$ represents free stream temperature 

The stream functions for the given flow problem are

(5) $$u=\frac{\partial \psi^{\prime }}{\partial y}\;\mathrm{and}\;v=-\frac{\partial \psi^{\prime }}{\partial x}.$$

Similarity transformations of the defined flow problem are

(6) $$\psi =\sqrt{\frac{a\nu_{f}}{1-\alpha t}}xf\left(\eta \right)\;\mathrm{And}\;\eta =y\sqrt{\frac{a}{v_{f}\left(1-\alpha t\right)}},\;\theta \left(\eta \right)=\frac{T-T_{\infty }}{T_{W}-T_{\infty }}.$$

Putting Equations (5) and (6) into Equations (1)–(4), gives

(7) $$f^{\prime \prime \prime }+{\left(1-\phi \right)}^{2.5}\left((1-\phi )+\phi \frac{\rho_{s}}{\rho_{s}}\right)\left[ff^{\prime \prime }-f\prime {}^{2}-S\left(f\prime +\frac{\eta }{2}f\prime \prime \right)\right]-{\left(1-\phi \right)}^{2.5}Mf^{\prime }=0\;$$

(8) $$\frac{k_{nf}}{k_{f}}\theta^{\prime \prime }-\left((1-\phi )+\phi \frac{{\left(\rho C_{p}\right)}_{CNT}}{{\left(\rho C_{p}\right)}_{f}}\right)\left(\mathrm{Pr}\left(\theta^{\prime }f\right)+\frac{s}{2}Ec\left(\eta \theta^{\prime }\right)\right)=0$$

With boundary conditions

(9) $$\begin{array}{l} f\left(\eta \right)=0,f^{\prime }\left(\eta \right)=\frac{c}{a}\;\;at\;\;\eta =0,f^{\prime }\left(\eta \right)\to 1\mathrm{as}\eta \to \infty ,\\ \theta \left(\eta \right)=1at\;\eta =0;\theta \left(\eta \right)\to 0\mathrm{as}\eta \to \infty . \end{array}$$

(10) $$\begin{array}{l} \frac{\rho_{nf}}{\rho_{f}}=(1-\phi )+\phi (\frac{\rho_{CNT}}{\rho_{f}}),\;\frac{\mu_{nf}}{\mu_{f}}={\left(1-\phi \right)}^{-2.5},\frac{{\left(\rho \;Cp\right)}_{nf}}{{\left(\rho \;Cp\right)}_{f}}=\left[\left(1-\phi )+\phi (\frac{{\left(\rho \;cp\right)}_{CNT}}{{\left(\rho \;cp\right)}_{f}}\right)\right],\\ \frac{k_{nf}}{k_{f}}=\frac{1-\phi +2\phi (\frac{k_{CNT}}{k_{CNT}-k_{f}})\;ln(\frac{k_{CNT}+k_{f}}{2\;k_{f}})}{1-\phi +2\phi (\frac{k_{f}}{k_{CNT}-k_{f}})\;\;ln(\frac{k_{CNT}+k_{f}}{2\;k_{f}})}. \end{array}$$

where prime denotes differentiation with respect to η and the constant a>0,b>0, $\mathrm{Pr}=\frac{\nu }{\alpha }$ is the Prandtl number. α is thermal diffusivity, $\mathrm{Ec}=\frac{u_{w}^{2}}{C_{p}\left(T_{w}-T_{\infty }\right)}$ represents the Eckert number, $S\;=\frac{\gamma }{b}\;,$ represents the unsteady parameter, ϕ represents a dimensionless nanoparticle volume friction, and $M=\frac{\sigma^{*}B_{0}^{2}}{\rho_{f}b}$ represents the magnetic field

### The Skin Friction and Nusselt Number 

In this analysis, the two key important quantities are skin friction and Nusselt number, given as

(11) Rex12Cnf=−1−ϕ−2.51−ϕ+ϕρ1ρff″(0),Rex−12Nux=−ksknfθ′(0), 

where Rex=U wL/ν ,.

## 3. Method of Solution

We apply the basic idea of OHAM to the given problem given below.

(12) $$f^{\prime \prime \prime }-ff\prime \prime -f\prime {}^{2}-f\prime \prime -f\prime =0,\;f\left(0\right)=0,\;f\prime \left(\infty \right)=1,\;f\prime \left(1\right)=\frac{c}{a}$$

where η is a similarity variable, $f\left(\eta \right)$ is related to the stream function and prime denote derivative with respect to η. Let λ>0 denote a kind of spatial scale parameter by means of transformation

(13) $$f\left(\eta \right)=\lambda^{-1}u\left(\xi \right),\;\xi =\lambda \eta$$

The original Equation (12) becomes

(14) $$\begin{array}{l} u\prime \prime \prime \left(\xi \right)-uu\prime \prime \left(\xi \right)-{\left(u\prime \left(\xi \right)\right)}^{2}-u\prime \prime \left(\xi \right)-u^{\prime }\left(\xi \right)=0\\ u\left(0\right)=0\;,u\prime \left(\infty \right)=1,\;u\left(1\right)=\frac{c}{a} \end{array}$$

The boundary condition $u^{\prime }\left(\infty \right)=1$, the asymptotic property u→ξ as ξ→∞. According to the theses information, $u\left(\xi \right)$ can be stated in the form

(15) $$u\left(\xi \right)=A_{0,0}+\xi +\sum_{m=1}^{\infty }\sum_{n=0}^{\infty }A_{m,n}\xi^{n}\exp\left(-m\xi \right)$$

where $A_{m,n}$ represent constants. The so called solution expression of $u\left(\xi \right)$ has an important role in OHAM. Subsequently, we have four boundary conditions, rendering to the solution (15) the simplest four terms of $u\left(\xi \right)$ are $A_{0,0},\xi ,A_{1,0}\exp\left(-\xi \right),A_{2,0}\exp\left(-\xi \right)$, we chose the initial solution in the form of

(16) $$u_{0}\left(\xi \right)=A_{0,0}+\xi +A_{1,0}\exp\left(-\xi \right)+A_{2,0}\exp\left(-\xi \right)$$

where $A_{0,0},A_{1,0},A_{2,0}$ are unknown constants. Enforcing $u_{0}\left(\xi \right)$ to satisfy the three boundary conditions, we have $A_{0,0}=0,A_{1,0}=-1,A_{2,0}=0$ so we have

(17) $$u_{0}\left(\xi \right)=\xi -1+e^{-\xi }$$

According to the solution (17), we should chose the linear operator L, in a manner that $L\left(u\right)=0$

(18) $$L\left(C_{0}+C_{1}\xi +C_{2}e^{-\xi }\right)$$

The estimation for velocity and for temperature are

(19) $$f_{0}\left(\eta \right)=\frac{c}{a}+e^{-\eta }$$

(20) $$\theta_{0}\left(\eta \right)=e^{-\eta }+1$$

which is obtained from the linear operator

(21) $$L_{f}=f\prime \prime \prime -ff\prime \prime =0\;\;,L_{\theta }=\;\theta \prime \prime =0,$$

## 4. Results and Discussion

**Figure 1:**
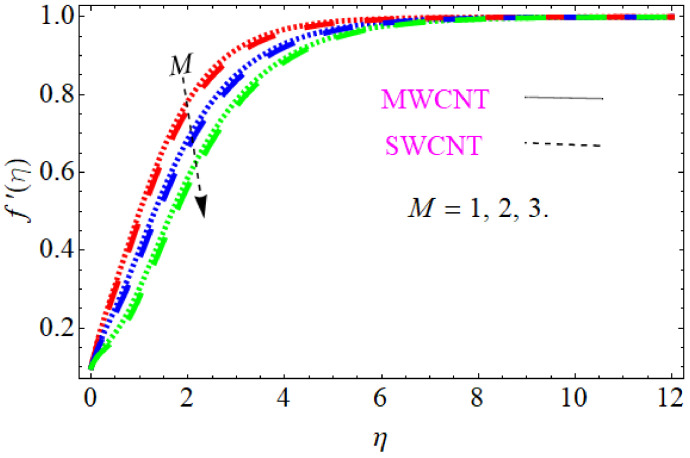
Consequence of M (magnetic field parameter) for velocity distribution.

**Figure 2:**
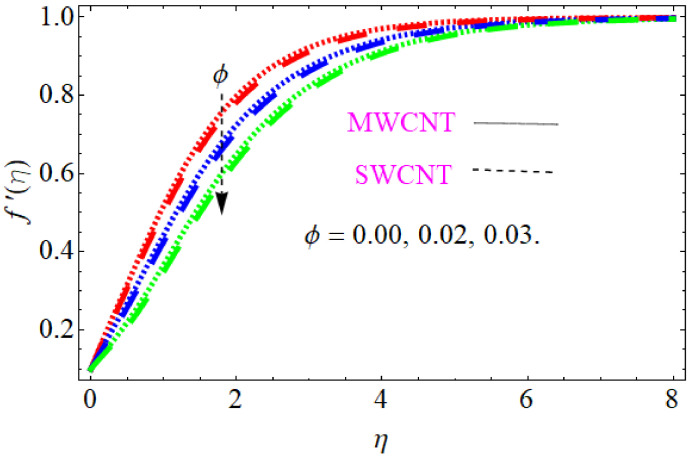
Consequence of dimensionless nanoparticle volume friction for velocity distribution.

**Figure 3:**
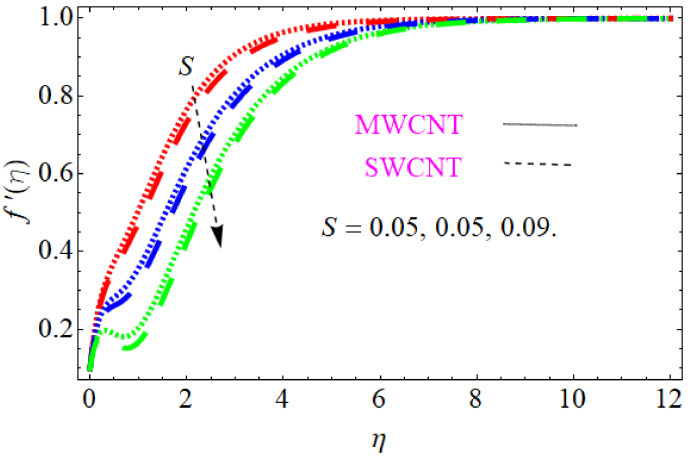
Consequence of unsteady parameter S for velocity distribution.

**Figure 4:**
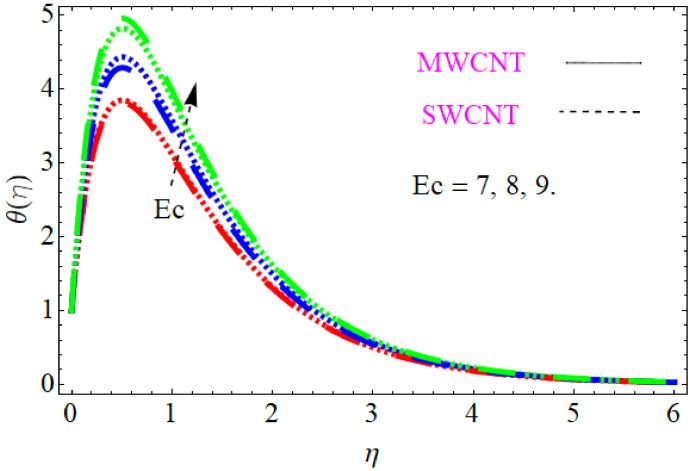
Consequence of the Eckert number for temperature distribution.

**Figure 5:**
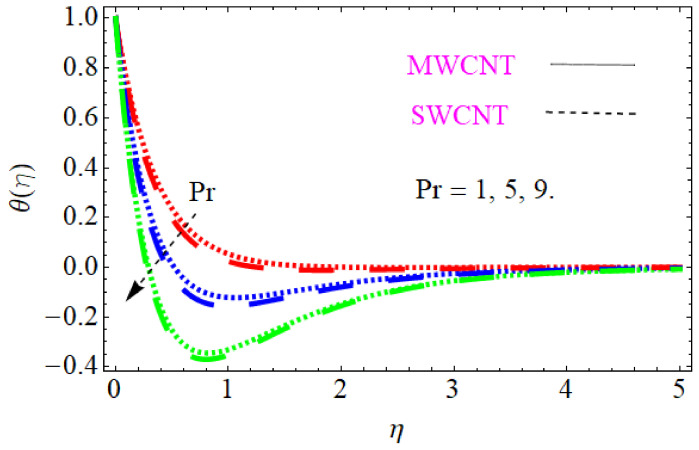
Consequence of Prandtl number for temperature distribution.

**Figure 6:**
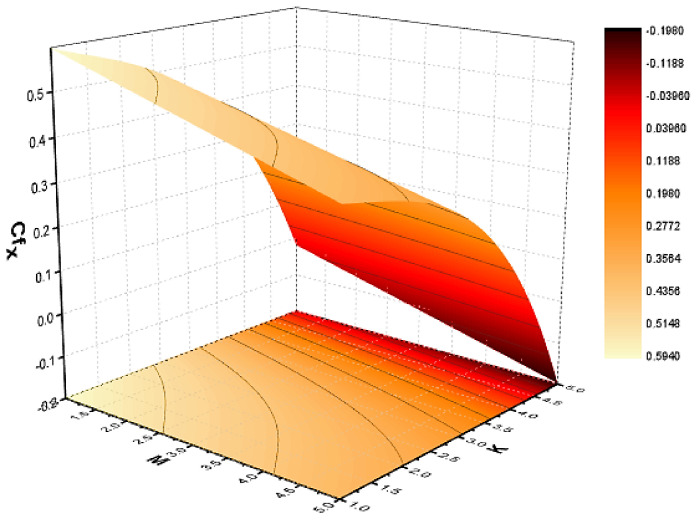
Effect of skin friction on M and S.

**Figure 7:**
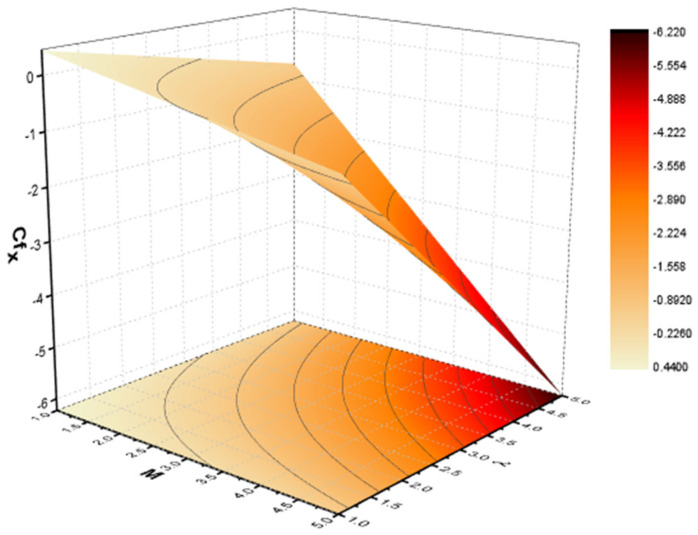
Effect on skin friction on M and ϕ.

**Figure 8:**
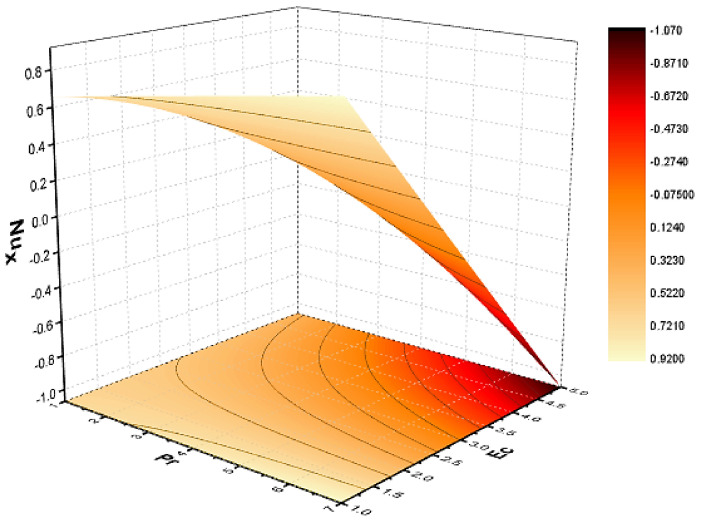
Effect of Nusselt number on Pr and Ec.

**Figure 9:**
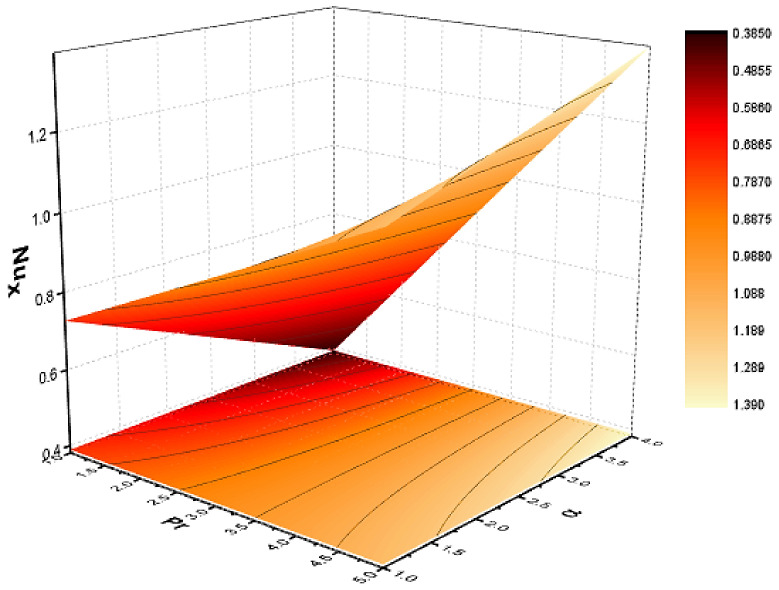
Effect of Nusselt number on Pr and M.

In this section, we discuss the influence of the different parameters, such as $\left\{M,S,\phi ,\mathrm{Pr},\mathrm{Ec}\right\}$ (magnetic field parameter, unsteady parameter, dimensionless nanoparticles volume friction, Prandtl number and Eckert number) on both $f\prime \left(\eta \right)\;and\;\theta \left(\eta \right)$. The CNT nanofluid is used for the enhancement of heat improvement applications. The flow investigation has studied on a movable surface, along with the magnetic field. To transform the nonlinear partial differential equation (PDE) to a nonlinear ordinary differential equation (ODE), we used the defined similarity transformation. The nonlinear differential equations were solved with the help of the approximate analytical method, named the optimal homotopy asymptotic method (OHAM). The convergence control parameter for the particular problems is also discussed in table form. With the help of the analytical method, a solution of the nonlinear differential equations for velocity and temperature profiles were obtained. The results of the particular problem are emphasized in Figure 1, Figure 2, Figure 3, Figure 4, Figure 5, Figure 6, Figure 7, Figure 8 and Figure 9; Figure 1, Figure 2 and Figure 3 represent the effect of dissimilar parameters on velocity distribution, Figure 4 and Figure 5 represent the effect of dissimilar parameters on temperature profile, Figure 6 and Figure 7 represent the effect of dissimilar parameters on $C_{f}$ (skin friction), and Figure 8 and Figure 9 represent the effect of dissimilar parameters on the Nusselt number. From Figure 6 and Figure 7 we can observe that skin friction is the function increasing the magnetic field parameter, unsteady parameter, and dimensionless nanoparticle volume friction; by increasing the magnitude of this parameter, resistance forces are produced which oppose the fluids particle motion, and due to this effect the skin friction increases. From Figure 8 and Figure 9, we can see that the Nusselt number is the increasing function of the Eckert number, Prandtl number, and magnetic field parameter; physically, by enhancing the magnitude of these parameters the fluid particles motion is reduced, and, as a result, friction forces are produced, due to this Nusselt number increasing. The convergence control parameter of the given problem was obtained up to the 25th iteration for both velocity and temperature distribution and presented in Table 1 and Table 2. From Table 1 and Table 2 we can see that as we increased the number of iterations, the residual error decreased, and a strong convergence was obtained on the approximate analytical solution. A comparison of the approximate analytical method to the numerical method is presented in Table 3 and Table 4. Table 5 and Table 6 represent the thermo-physical properties and thermo-conductivity of the nanofluids. The variation in magnetic field parameter M on the velocity profile is captured in Figure 1, for both MWCNT and SWCNT on $f\prime \left(\eta \right)$; from Figure 1 we see that velocity is the decreasing function of the magnetic field parameter. Such a state happens due to the creation of a resistive force known as the Lorentz force. The magnitude of these forces increases with the increase of the magnetic field parameter, which oppose the fluid particle motion in the opposite direction; the motion of the fluid particles decreases, and as a result the velocity profile decreases. The variation in the dimensionless nanoparticle volume fraction parameter ϕ on velocity profile is captured in Figure 2, for both the MWCNT and SWCNT velocity profile; from Figure 2 we can see that velocity is the decreasing function of the dimensionless nanoparticle volume fraction parameter ϕ. Such a state happens due to the production of a resistive type force, known as viscose force. The strength of such a force increase with the increase in strength of the dimensionless nanoparticle volume fraction parameter ϕ, which counteracts the motion of the fluid within the boundary layer and reduces the thickness of the boundary layer. The variation in the time-dependent parameter S in velocity profile is captured in Figure 3, and for both MWCNT and SWCNT we can see that velocity is the decreasing function of the time-dependent parameter S. Figure 4 shows the relation between Eckert number Ec and temperature profile; this relation is in direct relation, or the temperature profile is the increasing function of, Eckert number Ec. By increasing the Eckert number, this will improve the kinetic energy due to the intermolecular collision increasing, and, as a result, the temperature profile increases. Figure 5 shows the Pr for both MWCNT and SWCNT and the temperature distribution. It is noticeable from Figure 5 that the relation between $\theta \left(\eta \right)$ and Pr is an inverse relation, where the largest value of Pr reduces the temperature distribution. Actually the thickness of the momentum boundary layer is superior than that of the thermal boundary layer, or the viscous diffusion is larger than the thermal diffusion, and, therefore, a larger Prandtl number reduces the thermal boundary layer; therefore, the Prandtl number was used as the Colling agent.

## 5. Conclusions

This paper examined the analytical investigation of the stagnation point flow of a CNT nanofluid over a stretching surface. In this research article, we used an approximate analytical method for the solution of the nonlinear differential equation to find a series solution for velocity and another for temperature profile. To transform the nonlinear partial differential equation (PDE) into a nonlinear ordinary differential equation (ODE), we used the defined similarity transformation. By using the approximate analytical (OHAM) optimal homotopy asymptotic method to solve the obtained nonlinear ordinary differential equations. The impact of different parameters, such as magnetic field parameter, dimensionless nanoparticle volume fraction parameter, unsteady parameter, Prandtl number, and Eckert number were interpreted through graphs. The skin friction coefficient and Nusselt number were explained in the form of graphs. The present work was found to be in very good agreement with those published previously. The main outcomes of the present analysis are the following:

1. Increasing the value of the dimensionless nanoparticle volume fraction parameter reduces velocity.
2. Increasing the value of the magnetic field parameter reduces velocity
3. Increasing the value of the unsteady parameter reduces velocity
4. Increasing the value of the Prandtl number reduces the temperature profile
5. Increasing the value of the Eckert number increases the temperature profile

Of the numerous future physical world problems to be modeled in science and engineering, most of them will be nonlinear, so we cannot find the exact solution of the nonlinear differential equation for both the partial differential equation and ordinary differential equation; therefore, this method could be used in the future to find the approximate analytical series solution of the nonlinear differential equation, which has the best convergence to the exact solution. In the future, heat transfer phenomena will play a key role in engineering and industry. In our paper, we used a CNT nanofluid and discussed the heat transfer ratio, which plays an important role. 

## Figures and Tables

**Table 1:** Convergence of the method for MWCNT.

m	MWCNT	MWCNT
5	$0.1431\times 10^{-1}$	$0.4775\times 10^{-3}$
10	$0.3014\times 10^{-2}$	$0.7873\times 10^{-5}$
15	$0.3443\times 10^{-3}$	$0.4729\times 10^{-7}$
20	$0.9298\times 10^{-5}$	$0.5453\times 10^{-8}$
25	$0.3787\times 10^{-7}$	$0.2412\times 10^{-9}$

**Table 2:** Show the convergence of method for SWCNT.

m	SWCNT	SWCNT
5	$0.4391\times 10^{-1}$	$0.4574\times 10^{-1}$
10	$0.5166\times 10^{-3}$	$0.7359\times 10^{-2}$
15	$0.8238\times 10^{-5}$	$0.7749\times 10^{-5}$
20	$0.4626\times 10^{-6}$	$0.3121\times 10^{-7}$
25	$0.9433\times 10^{-9}$	$0.5132\times 10^{-9}$

**Table 3:** OHAM and numerical comparison for f(η).

m	Numerical	OHAM	Absolute Error
1	1.00….	1.00….	0.0….
2	1.72….	1.70….	$2\times 10^{-2}$….
3	1.71….	1.69….	$2\times 10^{-2}$….
4	1.96….	1.94….	$2\times 10^{-2}$….
5	0.87….	0.83….	$4\times 10^{-2}$….
6	0.23….	0.21….	$2\times 10^{-2}$….
7	0.29….	0.27….	$2\times 10^{-2}$….
8	0.39….	0.38….	$1\times 10^{-2}$….
9	0.42….	0.37….	$5\times 10^{-2}$….
10	0.92….	0.90….	$2\times 10^{-2}$….

**Table 4:** OHAM and numerical comparison for θ(η).

η	Numerical	OHAM	Absolute Error
1	1.00….	1.00….	0.0….
2	1.31….	1.29….	$2\times 10^{-2}$….
3	1.12….	1.10….	$2\times 10^{-2}$….
4	1.70….	1.65….	$5\times 10^{-2}$….
5	1.44….	1.41….	$3\times 10^{-2}$….
6	1.34….	1.30….	$4\times 10^{-2}$….
7	1.95….	1.90….	$5\times 10^{-2}$….
8	1.90….	1.80….	$1\times 10^{-2}$….
9	1.54….	1.50….	$4\times 10^{-2}$….
10	1.35….	1.30….	$5\times 10^{-2}$….

**Table 5:** The thermo-physical properties.

Physical Properties		Thermal Conduct K(W/mk)	$\mathrm{Specific}\;\mathrm{Heat}\;{}_{\mathrm{Cp}}(J/\mathrm{kgK})$	$\mathrm{Density}\;{\rho (\mathrm{kg}/m}^{3})$
Solid particles	SWCNTs	6600	2600	2600
	MWCNTs	3000	1600	1600

**Table 6:** The thermal conductivity values at different volume fractions.

Volume Fraction φ	0.0	0.01	0.02	0.03	0.04
$k_{\mathrm{nf}}\left(\mathrm{SWCNTs}\right)$	0.145	0.147	0.204	0.235	0.266
$k_{\mathrm{nf}}\left(\mathrm{MWCNTs}\right)$	0.145	0.172	0.2	0.228	0.2257

## Data Availability

All data used in this manuscript have been presented within the article.

## Nomenclature

x, y,	Cartesian coordinates
u, v,	Velocity components
U_w_, V_w_	Velocities of the stretching sheet
S	Time dependent parameter
T	Local Temperature
M	Magnetic field
Nu_x_	Local Nusselt number
pr	Prandtl number
T_w_	Surface temperature
B_0_	Constant magnetic field
T_∞_	Ambient temperature-
Ec	Eckert number
C_fx_	Skin friction coefficient in x− direction-
θ	Dimensionless temperature
ρ	Density of the fluid
ν	Fluid viscosity
η	Independent variable
λ	Spatial scale parameter
k	Thermal conductivity
$c_{p}$	Specific heat
